# Improving Clinical Risk Stratification at Diagnosis in Primary Prostate Cancer: A Prognostic Modelling Study

**DOI:** 10.1371/journal.pmed.1002063

**Published:** 2016-08-02

**Authors:** Vincent J. Gnanapragasam, Artitaya Lophatananon, Karen A. Wright, Kenneth R. Muir, Anna Gavin, David C. Greenberg

**Affiliations:** 1 Academic Urology Group, Department of Surgery, University of Cambridge, Cambridge, United Kingdom; 2 Institute of Population Health, University of Manchester, Manchester, United Kingdom; 3 Division of Health Sciences, Warwick Medical School, University of Warwick, Coventry, United Kingdom; 4 National Cancer Registration Service Eastern Office, Public Health England, Cambridge, United Kingdom; 5 Northern Ireland Cancer Registry, Centre for Public Health, Queen’s University Belfast, Belfast, United Kingdom; Harvard Medical School, UNITED STATES

## Abstract

**Introduction:**

Over 80% of the nearly 1 million men diagnosed with prostate cancer annually worldwide present with localised or locally advanced non-metastatic disease. Risk stratification is the cornerstone for clinical decision making and treatment selection for these men. The most widely applied stratification systems use presenting prostate-specific antigen (PSA) concentration, biopsy Gleason grade, and clinical stage to classify patients as low, intermediate, or high risk. There is, however, significant heterogeneity in outcomes within these standard groupings. The International Society of Urological Pathology (ISUP) has recently adopted a prognosis-based pathological classification that has yet to be included within a risk stratification system. Here we developed and tested a new stratification system based on the number of individual risk factors and incorporating the new ISUP prognostic score.

**Methods and Findings:**

Diagnostic clinicopathological data from 10,139 men with non-metastatic prostate cancer were available for this study from the Public Health England National Cancer Registration Service Eastern Office. This cohort was divided into a training set (*n* = 6,026; 1,557 total deaths, with 462 from prostate cancer) and a testing set (*n* = 4,113; 1,053 total deaths, with 327 from prostate cancer). The median follow-up was 6.9 y, and the primary outcome measure was prostate-cancer-specific mortality (PCSM). An external validation cohort (*n* = 1,706) was also used. Patients were first categorised as low, intermediate, or high risk using the current three-stratum stratification system endorsed by the National Institute for Health and Care Excellence (NICE) guidelines. The variables used to define the groups (PSA concentration, Gleason grading, and clinical stage) were then used to sub-stratify within each risk category by testing the individual and then combined number of risk factors. In addition, we incorporated the new ISUP prognostic score as a discriminator. Using this approach, a new five-stratum risk stratification system was produced, and its prognostic power was compared against the current system, with PCSM as the outcome. The results were analysed using a Cox hazards model, the log-rank test, Kaplan-Meier curves, competing-risks regression, and concordance indices. In the training set, the new risk stratification system identified distinct subgroups with different risks of PCSM in pair-wise comparison (*p <* 0.0001). Specifically, the new classification identified a very low-risk group (Group 1), a subgroup of intermediate-risk cancers with a low PCSM risk (Group 2, hazard ratio [HR] 1.62 [95% CI 0.96–2.75]), and a subgroup of intermediate-risk cancers with an increased PCSM risk (Group 3, HR 3.35 [95% CI 2.04–5.49]) (*p <* 0.0001). High-risk cancers were also sub-classified by the new system into subgroups with lower and higher PCSM risk: Group 4 (HR 5.03 [95% CI 3.25–7.80]) and Group 5 (HR 17.28 [95% CI 11.2–26.67]) (*p <* 0.0001), respectively. These results were recapitulated in the testing set and remained robust after inclusion of competing risks. In comparison to the current risk stratification system, the new system demonstrated improved prognostic performance, with a concordance index of 0.75 (95% CI 0.72–0.77) versus 0.69 (95% CI 0.66–0.71) (*p <* 0.0001). In an external cohort, the new system achieved a concordance index of 0.79 (95% CI 0.75–0.84) for predicting PCSM versus 0.66 (95% CI 0.63–0.69) (*p <* 0.0001) for the current NICE risk stratification system. The main limitations of the study were that it was registry based and that follow-up was relatively short.

**Conclusions:**

A novel and simple five-stratum risk stratification system outperforms the standard three-stratum risk stratification system in predicting the risk of PCSM at diagnosis in men with primary non-metastatic prostate cancer, even when accounting for competing risks. This model also allows delineation of new clinically relevant subgroups of men who might potentially receive more appropriate therapy for their disease. Future research will seek to validate our results in external datasets and will explore the value of including additional variables in the system in order in improve prognostic performance.

## Abstract 2

Vincent Gnanapragasam and colleagues test the performance of a new 5-category risk stratification scheme for primary prostate cancer, using data from large cohorts of men with localised or locally-advanced disease.

## Author Summary

**Why Was This Study Done?:**

- Prostate cancer incidence is rising worldwide, and, with improved detection, increasing proportions of men are presenting with non-metastatic disease (over 80%). Amongst these men, the disease is heterogeneous, and different management options are possible.
- Risk stratification is the primary method of deciding which treatment is appropriate for an individual. However, the current method of risk stratification is based on historical data and was not originally validated against prostate cancer mortality as an outcome. Moreover, no current risk stratification system has been developed first in an unscreened population, which represents the vast majority of men presenting with prostate cancer worldwide.
- Current risk models therefore require improvement to be more relevant for the management of prostate cancer in patients. In this study, we sought to improve clinical risk stratification by refining the attributes that make up the current risk stratification system and incorporating the latest pathological grading system for prostate cancer from the International Society of Urological Pathology.

**What Did the Researchers Do and Find?:**

- We studied a large dataset from a cohort of UK patients. Data from 10,139 men were available, and the cohort was split into a training group and a testing group for analysis.
- Clinico-pathological characteristics at diagnosis (including clinical stage, biopsy grade, and prostate-specific antigen [PSA] concentration) were used first to categorise patients according to the standard three-stratum risk stratification system (from the UK NICE guidelines). These same three individual characteristics were then used to sub-stratify within each risk group. In addition, we incorporated the new pathological prognostic grading system (score 1–5) recently adopted by the International Society of Urological Pathology.
- We found that the new risk model (with five subgroups) was significantly better at identifying patient populations with very different outcomes in terms of prostate-cancer-specific mortality. The model performance held true even when other competing risks of death were included. Most importantly, the model demonstrated improved prognostic power in comparison to the NICE stratification system, both in our primary cohort and in a separate external validation cohort.

**What Do These Findings Mean?:**

- To our knowledge, this study is the first to test the standard three-stratum risk stratification system in an unscreened first diagnosis population and to measure this system’s ability to predict prostate-cancer-specific mortality. We show that this model has a poor concordance for predicting mortality outcome at the point of diagnosis and is probably of little value in this context.
- Our new model performs much better and not only improves prediction of mortality but also provides better distinction of patient subgroups to inform clinical decision making. Moreover, the cohorts used for our study are more representative of real-world practice, where screening for prostate cancer is uncommon.
- These findings do need further validation in independent external cohorts, and our study is limited by its reliance on cancer registry records and relatively short follow-up.
- Nevertheless, the large sample size and the consistency of our findings in external validation suggest that these findings are robust and ready for clinical use. The new model does not require any additional variables other than those routinely collected at diagnosis in any clinic setting worldwide and will therefore be simple to adopt internationally.

## Introduction

Prostate cancer is one of the most common male cancers in the world, with nearly a million men diagnosed annually, and its incidence is increasing [1,2]. It is estimated that the number of new prostate cancer diagnoses in the UK alone will approach 70,000 per annum by 2030, an increase of 69% over the current incidence [3]. Of this population, over 80% will be men presenting with localised or locally advanced non-metastatic disease. This demographic, therefore, is and will increasingly become a major burden for healthcare systems internationally [4].

Risk estimation is the cornerstone of management for these men. The most widely used system is the three-stratum D’Amico classification first described in the late 1990s [5]. This original model has been widely adopted by different international guideline groups and organisations, although with some variations [6–8]. All, however, rely on the same clinico-pathological information available at diagnosis: the presenting prostate-specific antigen (PSA) concentration, the Gleason pathological grade sum, and clinical stage. This information is used to classify patients as low, intermediate, or high risk using specific cutoff values, which then guides treatment decisions [5–8]. It is now clear that within these standard groupings there exists significant heterogeneity in outcomes [9,10]. Key problems from this heterogeneity include the overtreatment of many indolent cancers and the converse issue of undertreatment of men with potentially aggressive disease. Recognising this, the International Society of Urological Pathology (ISUP) has recently adopted a prognosis-based pathological classification of prostate cancer based on a re-evaluation of the association of Gleason grading with treatment outcome [11]. This development is particularly welcome as work from our own centre and others has shown significant grade inflation in contemporary cohorts that is not necessarily linked to a poorer outcome [12,13].

A number of organisations have sought to improve risk stratification and identify subgroups with different outcomes. The US National Comprehensive Cancer Network has, for instance, included a very low-risk group (based on the percentage of positive biopsies) and a very high-risk group (based on clinical stage) in their guidelines [8]. The UCSF-CAPRA (University of California, San Francisco Cancer of the Prostate Risk Assessment) score in addition to the standard variables also considers the percentage of positive prostate biopsies (<34% or ≥34%) and patient age (<50 or ≥50 y) to improve prediction of risk [14]. The Genitourinary Radiation Oncologists of Canada (GUROC) group is currently exploring new risk models using recursive partitioning to better predict biochemical-relapse-free survival after radiotherapy [15]. Very importantly, however, none of these current risk models were originally developed in cohorts of newly diagnosed patients, nor do they use prostate-cancer-specific mortality (PCSM) as an outcome.

A novel approach to risk sub-stratification was recently reported by the EMPACT group in patients with high-risk surgically treated prostate cancer [16]. The EMPACT study demonstrated that better and poorer performing subgroups could be identified by considering the number of high-risk factors an individual had. In this current study, we explored whether this notion could be applied in other prostate cancer risk stratification systems and in the context of predicting prognosis in a primary diagnosis population. We also considered the impending changes in the pathological classification from ISUP. In the new classification, five prognostic groups will be defined and reported together with classical Gleason grading [17]. These prognostic groups have yet to be included and evaluated within any contemporary risk stratification system. Our goal was to test whether a new clinical risk stratification model could be developed that would provide a better predictive model for PCSM at the point of first diagnosis.

## Methods

### Study Cohorts

Primary prostate cancers (ICD-10 code C61) diagnosed in residents of the East of England Cancer Network area between 1 January 2000 and 31 December 2010 were registered by the Public Health England National Cancer Registration Service Eastern Office (NCRS[E]). This area covers 2.67 million people (1.32 million men). Recent reports have highlighted the completeness of information at NCRS(E) [18]. Primary sources of information included electronic and paper-based reports, clinical notes, and pathology results from ten hospitals, of which only two were academic centres. The data are therefore closely representative of real-world contemporary clinical practice. Cases with any metastatic involvement (as documented by M stage disease and/or positive bone or CT scan) were excluded. The stage assigned to each tumour was an integrated TNM stage (fifth edition up to 2009 and seventh edition in 2010) at diagnosis and was assigned by the NCRS(E) consultant oncologist and/or consultant histopathologist based on combined clinical, imaging, and pathological information. Subdivision within each T stage was available for only a minority of cases; hence, stage was ascribed as T1, T2, T3, or T4 only. Similarly, there were no data available on the number or percentage of core involvement from diagnostic biopsies as this parameter is not recorded in registry data. Electronic death notifications were received from the Office for National Statistics. Vital status was also checked using the Health and Social Care Information Centre Personal Demographics Service batch tracing system (http://systems.hscic.gov.uk/demographics/pds/). Cause of death was classified as prostate cancer specific when listed in cause 1[a], 1[b], or 1[c] of the death certificate, except when a cancer with a markedly worse prognosis was listed in cause 1[a]. Survival times were calculated from the date of diagnosis to date of death (prostate cancer specific and all cause) or date of censoring (30 September 2013). The median follow-up was 6.9 y for the primary cohort. Only cases with all components of diagnostic stage, primary and secondary grade, and presenting PSA (ng/ml) as well as data on follow-up and survival were included as these variables were essential to build the risk model. Any cases where these data were missing were therefore not included. The final primary cohort used for the testing and training sets therefore comprised 10,139 individuals in total, with 789 prostate cancer deaths and 2,610 overall deaths. To validate the results, we sourced an available independent dataset from the Northern Ireland Cancer Registry, which has information on all population PSA tests linked to prostate cancer diagnosis and death covering a similar time period and with all data fields available as above. This cohort comprised 1,706 individuals, with 43 prostate cancer deaths, 144 all-cause deaths, and a median follow up of 4.8 y.

### Statistical Analysis

Using the cohort of 10,139 men, we used a split-sample validation method to develop and test the model, with random seed number generation to divide the cohort into 60% (*n* = 6,026; 1,557 total deaths, with 462 from prostate cancer) as a training set and 40% (*n* = 4,113; 1,053 total deaths, with 327 from prostate cancer) as a separate validation testing set. The primary outcome of interest in this study was PCSM. Patients were initially categorised as low, intermediate, or high risk based on the UK National Institute for Health and Care Excellence (NICE) risk stratification system, which is itself derived from the widely accepted D’Amico classification [5,7]. The individual variables used to define the groups (PSA concentration, Gleason pathological grade sum, and clinical stage) were then used to sub-stratify within each risk category by testing the individual and then combined effect of the number of risk factors.

In addition, we used the proposed new ISUP prognostic score as a discriminator [17]. Notable features of the new scoring system are the independent value of a Gleason 4 + 3 histological diagnosis (ISUP prognostic score 3) and sub-classification of Gleason sum 8 and 9–10 (ISUP prognostic scores 4 and 5, respectively). Based on this, we derived a new risk stratification system that identified five potential outcome groups for PCSM. This model was then compared against the NICE stratification system in the training set. To compare survival differences between each risk group, we applied a Cox hazards model and the log-rank test with pair-wise comparisons. This analysis was then repeated for the testing set. The null hypothesis was no difference between risk groups in the probability of an outcome event (prostate cancer death) at any time point. For visual comparison and to explore estimation of survival time for each risk group, Kaplan-Meier plots with 95% confidence intervals are presented for both training and testing sets. Visual calibration plotting across the risk groups for both the NICE and the new risk stratification system was also performed for the testing set by using Cox estimates at 10 y of follow-up. To test whether primary treatment modality had any effect on group survival outcomes, the Mantel-Haenszel test was used to compare the incidence rate ratios of radical therapy uptake. Competing-risks regression using the Fine-Gray test was applied to include the potential influence of non-cancer deaths on the performance of the model. Hazard ratios (HRs) were generated and compared between the risk groups in the testing set.

For model discrimination, we used the somersd package in STATA to compute the rank parameters concordance index. Sub-distribution HRs were used instead of HRs in the computation in the competing-risks analysis [19]. We then compared the performance of the new model to the NICE stratification system. Concordance index comparison was also applied to the external validation cohort. For model calibration with inclusion of competing risks, we used a visual calibration plot across the risk groups for both the NICE and the new risk stratification systems for the testing set. We plotted observed and predicted survival probability at 10 y for each of the risk groups. The STATA command stcrprep was used, which allows fitting of Fine-Gray models [20]. A difference was considered statistically significant when the *p*-value was less than 0.05. All data were anonymised at source in the NCSR(E) before being used for analysis, and as a result no formal ethics review was deemed necessary for this study. All statistical analyses were performed using STATA/MP version 12.1. The analysis for this study was not preplanned, but we have included the analysis steps in S1 Text.

## Results

### Cohort Description and New Risk Stratification System Comparison

**Fig 1:**
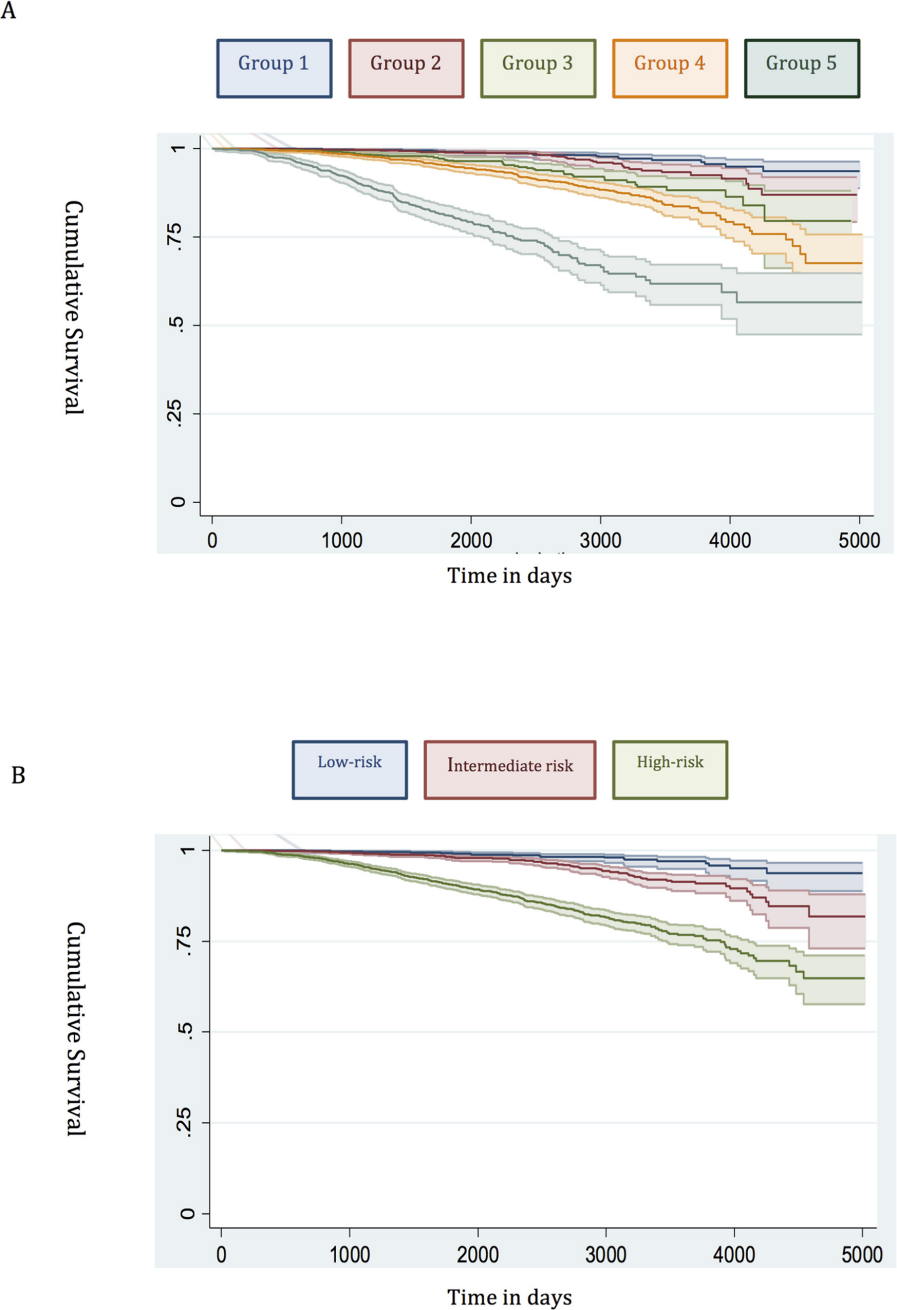
Survival stratified by risk group in the training set. (A) New risk stratification system applied to training set. (B) NICE risk stratification system applied to training set. Kaplan-Meier curves for prostate-cancer-specific survival and 95% confidence intervals (shaded areas) are shown for each risk group (*n* = 6,026).

**Fig 2:**
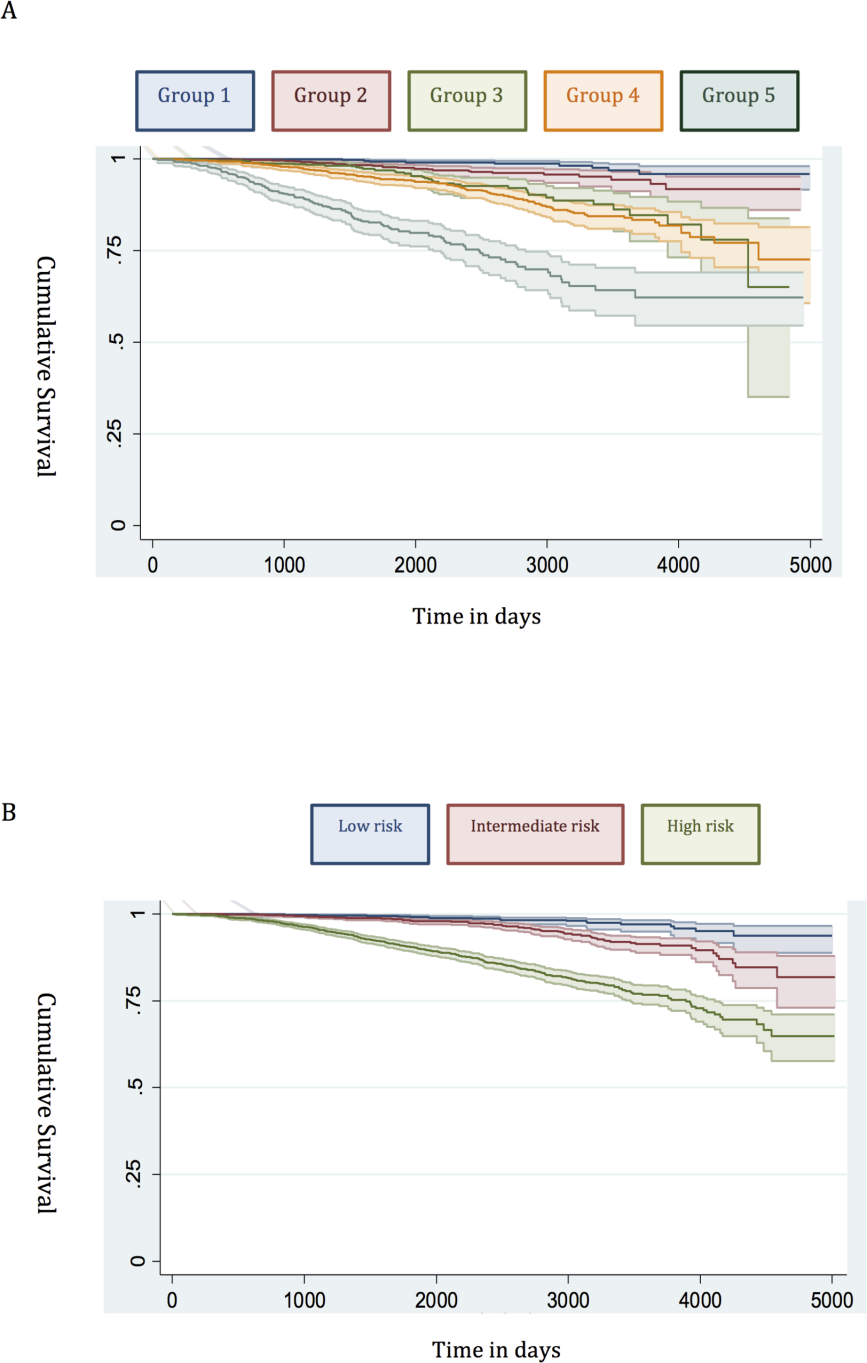
Survival stratified by risk group in the testing set. (A) New risk stratification system applied to testing set. (B) NICE risk stratification system applied to testing set. Kaplan-Meier curves for prostate-cancer-specific survival and 95% confidence intervals (shaded areas) are shown for each risk group (*n* = 4,113).

**Fig 3:**
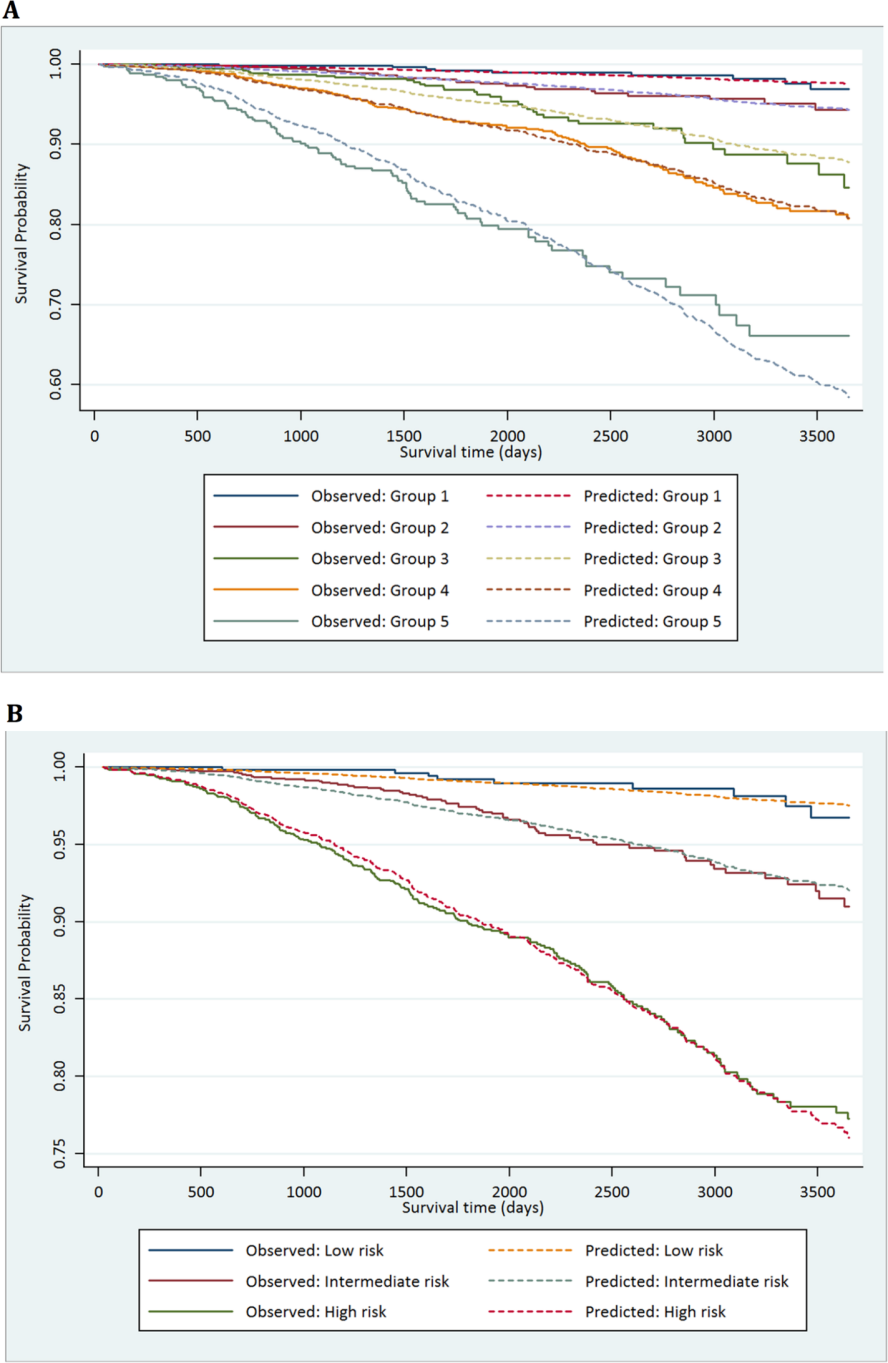
Calibration plots of the risk groups. (A) Calibration curves for prostate-cancer-specific survival using the new risk stratification system applied to the testing set (*n* = 4,113). (B) Model calibration curves for prostate-cancer-specific survival using the NICE risk stratification system applied to the testing set.

The demographic details of the primary study cohort are shown in Table 1. There was a good distribution across all clinico-pathological characteristics, and the distributions were representative of contemporary disease presentation trends in the UK [3]. From this cohort, five new risk groups were defined as described in the Methods (Table 2). These groups corresponded to sub-classification of both intermediate-risk and high-risk cancers but maintained the current definition of low-risk disease. In both the training and testing sets, Kaplan-Meier curves confirmed distinct prostate cancer survival outcomes for the risk groups using the new stratification system (Figs 1 and 2). The comparable prostate cancer survival curves of the current NICE stratification system are also shown in Figs 1 and 2. Data on the distribution of cases and deaths as well as HRs for each of the new risk groups for both sets are shown in S1 and S2 Tables. Visual calibration plots showed that both the new risk model and the NICE stratification system were well calibrated in the testing set for each risk group (Fig 3). Finally, we also tested for any differences in uptake of radical therapy between the new risk groups, which might have influenced survival outcome. The breakdown of different treatments for each new risk group is shown in S3 Table. In a test for homogeneity, the incidence rate ratios for radical therapy (surgery, radiotherapy, or brachytherapy) were not different between the new risk groups (S4 Table).

**Table 1:** Distribution of the primary study cohort (*n* = 10,139) by age, PSA at presentation, biopsy grade, and clinical stage.

Clinico-pathological Characteristic	n
**Age (y)**	
<60	1,121
60–69	3,717
70–79	4,012
≥ 80	1,289
**PSA (ng/ml)**	
<10	4,118
10–20	3,306
>20	2,715
**Biopsy grade/ISUP prognostic score**	
≤3 + 3/prognostic score 1	3,411
3 + 4/prognostic score 2	2,991
4 + 3/prognostic score 3	1,503
8/prognostic score 4	1,004
9–10/prognostic score 5	1,230
**Stage**	
T1	5,452
T2	3,226
T3	1,384
T4	77

**Table 2:** Proposed new prostate cancer risk stratification system.

New Risk Group	Criteria
1	Gleason 6 (prognostic score 1) **AND** PSA < 10 ng/ml **AND** Stage T1–T2
2	Gleason 3 + 4 = 7 (prognostic score 2) **OR** PSA 10–20 ng/ml **AND** Stage T1–T2
3	Gleason 3 + 4 = 7 (prognostic score 2) **AND** PSA 10–20 ng/ml **AND** Stage T1–T2 **OR** Gleason 4 + 3 = 7 (prognostic score 3) **AND** Stage T1–T2
4	Any one of Gleason 8 (prognostic score 4) **OR** PSA > 20 ng/ml **OR** Stage T3
5	More than one of Gleason 8 (prognostic score 4), PSA > 20 ng/ml, Stage T3 **OR** Any Gleason 9–10 (prognostic score 5) **OR** Any Stage T4

The prognostic scores refer to the new ISUP classification [11].

### Risk Group Sub-classification

#### Low risk

Many studies have already shown that the traditional D’Amico low-risk group identifies men with a very low incidence of prostate cancer mortality [12,21,22]. In our new system, Group 1 encompassed all low-risk cancers, and the prostate cancer mortality rate was correspondingly very low (Fig 1). This was seen in both the training and testing sets (Figs 1 and 2). Attempts to further sub-classify low-risk cancers using different PSA cutoffs or separating T1 and T2 tumours did not lead to any better or worse performing group. Thus, we conclude that low-risk cancers already represent a very indolent form of tumour, and further sub-stratification is unlikely to be useful, at least using clinical parameters alone.

#### Intermediate risk

Intermediate-risk cancers represent the largest and most heterogeneous group in contemporary practice [8]. In the primary cohort alone, over 37% of the population was intermediate risk (by the NICE stratification system). For our new stratification system, we hypothesised that a combination of intermediate-risk factors and/or primary Gleason 4 (ISUP prognostic group 3) alone might confer a greater risk of prostate cancer mortality (Table 2). In the training set, we observed that men within Group 3 (which included these factors) had a much worse outcome compared to men with a single intermediate-risk factor (Group 2): HR 3.35 (95% CI 2.04–5.49) versus 1.62 (95% CI 0.96–2.75), *p <* 0.0001 (Fig 1; S1 Table). This was further illustrated in a pair-wise comparison whereby Groups 2 and 3 represented distinctly different cohorts with regards PCSM (*p <* 0.0001) (Table 3). Men with only a single intermediate-risk factor (Group 2) had a very favourable prognosis, with outcomes approaching those of the lowest risk group in the study (Fig 1; S1 Table). These results were recapitulated in the testing set, in that intermediate-risk cancers could be reclassified into two distinct groups with very different HRs and outcomes: Group 3 HR 5.49 (95% CI 2.75–10.96) versus Group 2 HR 2.42 (95% CI 1.17–4.98), *p <* 0.0001 (Fig 2; Tables S2 and 4).

**Table 3:** Log-rank pair-wise comparison of new risk groups’ association with prostate-cancer-specific mortality in the training set (*n* = 6,026).

New Risk Group	New Risk Group							
	1		2		3		4	
	X^2^	*p*-Value	*X* ^2^	*p*-Value	*X* ^2^	*p*-Value	*X* ^2^	*p*-Value
1	—	—						
2	4.21	0.040	—	—				
3	27.25	<0.0001	12.45	<0.0001	—	—		
4	66.36	<0.0001	40.83	<0.0001	5.72	0.017	—	—
5	262.47	<0.0001	239.39	<0.0001	127.09	<0.0001	140.08	<0.0001

**Table 4:** Log-rank pair-wise comparison of new risk groups’ association with prostate-cancer-specific mortality in the testing set (*n* = 4,113).

New Risk Group	New Risk Group							
	1		2		3		4	
	X^2^	*p*-Value	*X* ^2^	*p*-Value	*X* ^2^	*p*-Value	*X* ^2^	*p*-Value
1	—	—						
2	6.30	0.012	—	—				
3	33.30	<0.0001	12.64	<0.0001	—	—		
4	48.27	<0.0001	28.14	<0.0001	1.53	0.216	—	—
5	157.16	<0.0001	148.86	<0.0001	67.80	<0.0001	79.79	<0.0001

#### High risk

In the training set, we found that a combination of high-risk factors or ISUP prognostic score 5 (Gleason grade 9–10) conferred the greatest risk of prostate cancer mortality, with a HR of 17.28 (95% CI 11.2–26.67) (Fig 1; S1 Table). This group also included T4 disease, but the contributing numbers were small (*n* = 77 in the whole cohort). Conversely, having a single high-risk factor of Gleason grade 8 (ISUP prognostic score 4) or PSA > 20 ng/ml or stage T3 conferred a much better outcome, with a HR of 5.03 (95% CI 3.25–7.80). These differences were clearly illustrated in a pair-wise comparison test where Groups 4 and 5 represented statistically different groups (Table 3) (*p <* 0.0001). These findings were recapitulated in the testing cohort. Here, the HRs were 21.56 (95% CI 11.52–41.07) for Group 5 and 7.05 (95% CI 3.69–13.46) for Group 4, respectively (*p <* 0.0001) (Fig 2; S2 Table). Pair-wise comparison further illustrated the difference in outcome between the two groups (Table 4). These results suggest a clear distinction in mortality outcomes within the traditional grouping of high-risk disease that can be identified at diagnosis.

#### Competing-Risks Analysis

**Fig 4:**
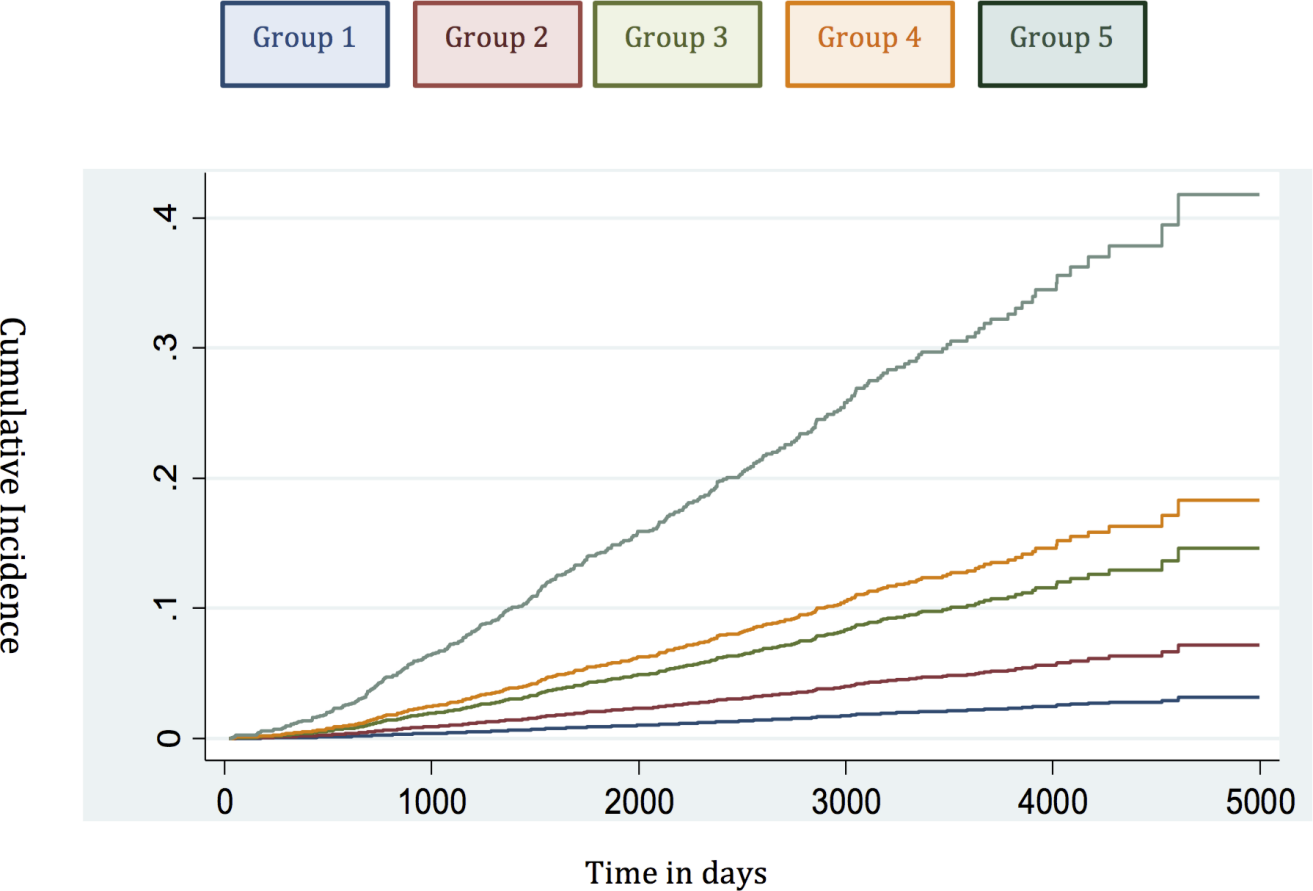
Cumulative incidence curves applied in the testing set to assess the competing mortality risks in the new model.

To account for deaths from other causes, we next carried out competing-risks analysis in the testing set to see whether this had any effect on the HRs of our new model. In this analysis, the results continued to demonstrate a good separation in survival outcomes for the new risk model in intergroup comparisons and cumulative incidence curves (Table 5; Fig 4). A log likelihood ratio test was also significantly different in favour of the new risk groups in the testing set (*p <* 0.0001), suggesting that the new risk model provided a better overall fit than the NICE stratification system. Calibration curves incorporating competing risks in the testing set further demonstrated a good calibration of our model for all groups (S1 Fig). These data suggest that inclusion of competing risks does not adversely affect the performance of our new risk model in discriminating subgroups with different prostate cancer survival outcomes.

**Table 5:** Competing-risks regression analysis of the new risk model in the testing set.

Risk Group Comparison	HR	95% CI	*p*-Value
1 versus 2	2.35	1.15–4.81	0.019
2 versus 3	2.18	1.35–3.52	0.001
3 versus 4	1.51	1.08–2.13	0.017
4 versus 5	2.24	1.73–2.89	<0.0001

Intergroup comparisons are shown, demonstrating clear differences in outcome between groups.

### Concordance Index Comparison

Our analysis has shown that the proposed new stratification system is able to identify subgroups of men within each classical risk category (except in low-risk cancer) with distinctly different PCSM outcomes. A crucial question therefore is whether this translates into a better prognostic model for PCSM compared to D’Amico-classification-based stratification systems such as that recommended by NICE. To assess this, we compared the concordance index of the new risk model and the NICE stratification system in the testing set with incorporation of competing mortality risks. In this cohort, the three-stratum NICE risk stratification system had a concordance index of 0.69 (95% CI 0.66–0.71) for predicting PCSM (Table 6). The new risk stratification system, in comparison, had an improved index of 0.75 (95% CI 0.72–0.77) (*p <* 0.0001 for model discrimination). To further test the prognostic improvement of our new model, we sourced an independent dataset from the Northern Ireland Cancer Registry. Details of the cohort are shown in S5 Table. In this cohort, the NICE concordance index was 0.66 (95% CI 0.63–0.69) (Table 6). The new risk stratification system concordance index however was higher, at 0.79 (95% CI 0.75–0.84) (*p <* 0.0001) (Table 6). We interpret this validation result with caution as this external cohort had a much shorter follow-up (4.8 y) and many fewer death events (43 cancer deaths and only 144 overall deaths). Nevertheless, the fact that our new model continued to show improved predictive power in this independent population compared to the NICE stratification system is encouraging.

**Table 6:** Concordance indices of the NICE stratification system and the new risk model for prostate-cancer-specific mortality, with inclusion of competing risks, in the testing cohort and external validation cohort (*p <* 0.0001 for both comparisons).

Cohort (*n*)	Concordance Index (95% CI)	
	NICE Stratification System	New Risk Model
Testing set (4,113)	0.69 (0.66–0.71)	0.75 (0.72–0.77)
Validation cohort (1,706)	0.66 (0.63–0.69)	0.79 (0.75–0.84)

## Discussion

This study has demonstrated a new stratification system that outperforms the standard three-stratum risk stratification system in predicting the risk of PCSM at diagnosis in men with primary non-metastatic prostate cancer. A key reason for the success and widespread adoption of the existing three-stratum system was its simplicity, based as it is on data routinely available at diagnosis. As a result, this paradigm has remained essentially unchanged for two decades and remains the first tool used by clinicians in counselling patients.

The primary clinical application of risk stratification is at the point of diagnosis, for treatment recommendation and predicting prognosis [6–8]. There have not, however, been any risk stratification systems that have been initially derived using this population. The original D’Amico classification was derived from just over 1,500 US men treated by surgery and radiotherapy, and used biochemical relapse as the primary outcome [5]. Other risk stratification systems and nomograms have also been built using data on outcomes after surgery or radiotherapy [23–25]. The closest comparator to our current system is the UCSF-CAPRA score. Originally derived from a series of 10,000 radical prostatectomies, its prognostic utility was retested in a US-registry-based cohort of 10,627 men who received different therapies and with a median follow-up only 5.9 y [26]. The score achieved good concordance in predicting metastasis and cause-specific mortality for every point increase in the calculated risk score (range 1–10). The source cohort, however, was a highly screened population, and, as a result, over 80% of new diagnoses were low to low-intermediate grade (Gleason 3 + 3/3 + 4), and 97% had organ-confined T1–T2 disease. Moreover, over 50% of the cases were treated surgically, and only 6% were managed by active surveillance/watchful waiting. This cohort is therefore very different to contemporary unscreened populations (e.g., in the UK) where 15%–20% of men present with locally advanced (T3–T4) disease, and conservative management is used in 14%–20% of men [7,27]. The UCSF-CAPRA score also requires data on the percentage of biopsy core involvement, which is not routinely recorded or standardised outside the US. Considering these factors, unique features of the risk stratification system proposed in this paper are its derivation from an unscreened primary diagnostic cohort (14% presenting with locally advanced T3–T4 disease) and that it encompasses all treatment types, including a significant proportion of men managed conservatively (19.7% in the primary cohort). Thus, our model is more representative of a contemporary, real-world unscreened primary diagnosis population.

The proposed risk stratification system identifies five risk groups and may better lend itself to informing therapy choices, though this awaits future evaluation. As an example, our new model subdivides the previous intermediate-risk and high-risk groups into two further subgroups with different survival outcomes. These subgroups could potentially be used to better determine which men should receive primary radical therapy or to stratify patients for treatment intensification trials. Of note, the current NICE guidelines are already used to direct recommendations for therapy, despite the fact that they were not originally intended for use in non-radical-therapy options nor calibrated against PCSM [7]. An intriguing question is whether our new risk stratification system might help select the best first radical treatment for an individual. Preliminary analysis suggests that the new system currently cannot preferentially show the benefit of one therapy over another. The addition of refinements, e.g., biopsy content and/or genomic markers, might allow this functionality in future.

There are inherent limitations in this study. It is based on registry records rather than a national prospective study. We also did not have central pathology review. This study necessarily included only men with intact data on stage, PSA concentration, and histological grade (to be able to construct the individual risk assignments), and this may have biased our results. Our follow-up is also relatively short in the context of prostate cancer, and we do plan to report the ongoing and maturing outcome of this cohort. This is particularly important for assessing any changes in PCSM in the lower risk groups. The cohort of patients in this study received different treatments, and the pattern of treatment use has changed through the decade [27]. We therefore cannot exclude the possibility that this might have biased survival outcomes, particularly in the highest risk group (e.g., inadequate therapy early in the series with androgen deprivation treatment alone and without radical therapy). However, we did test for the relative rates of radical curative therapy use and found no difference in proportional uptake in any group. Stage data in this study were limited to organ-confined (T1–T2) and locally advanced (T3–T4) categories, and sub-classifications were not available. It is, however, doubtful how much more stratification clinical stage subdivision would offer, as clinical staging is known to often be inaccurate [28,29]. Similarly, we did not have access to information on biopsy core involvement from pathology reports. We note, however, that none of the other current risk stratification systems based on national and international guidelines include the extent of core involvement in all of their risk group assignments [6–8].

The recent advent of multiparametric MRI of the prostate and image-guided biopsies will no doubt vastly improve the preciseness of diagnostic staging and the assessment of biopsy core involvement and, in turn, improve the accuracy of our risk stratification system. Finally, although we have used a relatively large training and testing set as well as an independent external cohort, our model does need further validation in independent cohorts with more death events and longer follow-up, and this is currently being explored. Our model was also not tested in this report for its ability to predict all-cause mortality compared to the current stratification system. Further data points, including the addition of age stratification and co-morbidity scores, may be needed for this purpose, and we will seek to address this in future refinements of the stratification system and using larger cohorts. It is however noteworthy that in competing-risks regression analyses, other causes of mortality did not alter the improved predictive performance of our new risk stratification system.

In summary, we present here an improved but simple risk stratification system for prostate cancer mortality in primary non-metastatic prostate cancer. This system is also, to our knowledge, the first reported model to incorporate the new ISUP prognostic score (which will be adopted by the World Health Organization in 2016). Importantly, we have not here sought to construct an individualised prognostic tool; instead, we have looked to build on and refine the most widely applied and clinically useful contemporary tool in risk-stratified management of prostate cancer. Our key findings are the following: (i) reinforcement of the indolent nature of the current low-risk category, (ii) identification of a subgroup of intermediate-risk men with a favourable prognosis, (iii) evidence that men with a combination of intermediate-risk factors or a single high-risk factor may have a very similar natural history, and (iv) identification of a subgroup of high-risk men with a particularly poor outcome.

Our results need further validation but may already be useful in contemporary clinical practice in better stratifying patients at the point of diagnosis. We further propose that this model is not static but provides an important new baseline to which additional prognostic information can be added, such as patient age, co-morbidity, biopsy core involvement, functional MRI characterisation, and molecular sub-typing. In this last regard, a number of genomic classifiers have been proposed and have shown some promise in improving prognostic prediction at diagnosis [30,31]. These molecular tests are commonly based on the premise that clinical risk stratification systems are already at the limit of prognostic power. As we have seen in this study, this is not necessarily the case. Our new system, evaluated in nearly 12,000 men and using routinely available clinical variables, had a high predictive ability for PCSM (concordance indices of 0.75–0.79), which is very similar to the published performance of genomic marker panels [32–34]. Our ongoing research will test how the new model can be further improved with the inclusion of these additional parameters. In the meantime, our new risk stratification system already shows improved prognostic utility compared to the current NICE stratification system in predicting the risk of prostate cancer death for men newly diagnosed with prostate cancer.

## Supporting Information

S1 Fig Model calibration curve of the new risk model incorporating competing mortality risks applied to the testing set. (TIFF)

S1 Table Distribution of cases/deaths and hazard ratios for each new risk group in the training set (*n* = 6,026). (DOCX)

S2 Table Distribution of cases/deaths and hazard ratios for each new risk group in the testing set (*n* = 4,113). (DOCX)

S3 Table Uptake of different treatment types in each new risk group across the whole cohort. (DOCX)

S4 Table Comparison of radical therapy use in each new risk group across the whole cohort. (DOCX)

S5 Table Distribution of the external validation cohort (*n* = 1,706) by age, PSA at presentation, biopsy grade, and clinical stage. (DOCX)

S1 Text Analysis plan. (DOCX)

S2 Text TRIPOD checklist. (DOCX)

## Abbreviations

HR: hazard ratio
ISUP: International Society of Urological Pathology
NCRS(E): National Cancer Registration Service Eastern Office
NICE: National Institute for Health and Care Excellence
PCSM: prostate-cancer-specific mortality
PSA: prostate-specific antigen
