# Supplementary material for: Improving Clinical Risk Stratification at Diagnosis in Primary Prostate Cancer: A Prognostic Modelling Study
Source: PLoS Med. 2016 Aug 2;13(8):e1002063. doi: 10.1371/journal.pmed.1002063 (PMC4970710; doi:10.1371/journal.pmed.1002063)
Supplement: S1 Table — (DOCX) [file pmed.1002063.s002.docx]

**Table S1** - Distribution of cases/deaths and hazard ratios for each new risk group category in the training set (n= 6026). Deaths refer to prostate cancer specific mortality.

|  |  |  |  |
| --- | --- | --- | --- |
| **New risk group** | **Number of men (deaths)** | **Hazard Ratio (95% CI)** | **p value** |
|  |  |  |  |
| **1** | 1087 (23) | 1 | NA |
| **2** | 1201 (35) | 1.62 (0.96-2.75) | 0.07 |
| **3** | 971 (50) | 3.35 (2.04-5.49) | <0.0001 |
| **4** | 1815 (156) | 5.03 (3.25-7.80) | <0.0001 |
| **5** | 952 (198) | 17.28 (11.20-26.67) | <0.0001 |
|  |  |  |  |
