# Supplementary material for: Improving Clinical Risk Stratification at Diagnosis in Primary Prostate Cancer: A Prognostic Modelling Study
Source: PLoS Med. 2016 Aug 2;13(8):e1002063. doi: 10.1371/journal.pmed.1002063 (PMC4970710; doi:10.1371/journal.pmed.1002063)
Supplement: S2 Table — (DOCX) [file pmed.1002063.s003.docx]

**Table S2** - Distribution of cases/deaths and hazard ratios for each new risk group category in the testing set (n= 4113). Deaths refer to prostate cancer specific mortality.

|  |  |  |  |
| --- | --- | --- | --- |
| **New risk group** | **Number of men (deaths)** | **Hazard Ratio (95% CI)** | **p value** |
|  |  |  |  |
| **1** | 653 (10) | 1 | NA |
| **2** | 877 (28) | 2.42 (1.17-4.98) | 0.017 |
| **3** | 666 (42) | 5.49 (2.75-10.96) | <0.0001 |
| **4** | 1237 (112) | 7.05 (3.69-13.46) | <0.0001 |
| **5** | 680 (135) | 21.56 (41.06) | <0.0001 |
|  |  |  |  |
