# Supplementary material for: Improving Clinical Risk Stratification at Diagnosis in Primary Prostate Cancer: A Prognostic Modelling Study
Source: PLoS Med. 2016 Aug 2;13(8):e1002063. doi: 10.1371/journal.pmed.1002063 (PMC4970710; doi:10.1371/journal.pmed.1002063)
Supplement: S3 Table — (DOCX) [file pmed.1002063.s004.docx]

**Table S3** – Uptake of different treatments types in each new risk group across the whole cohort. Conservative management includes patients managed by any non-interventional approach (active surveillance/watchful waiting). PADT – Primary androgen deprivation therapy.

| **Treatment type** | **Group 1**  (n=1740) | **Group 2**  (n=2078) | **Group 3**  (n=1637) | **Group 4**  (n=3052) | **Group 5**  (n=1632) |
| --- | --- | --- | --- | --- | --- |
| **Conservative management** | 812 | 633 | 306 | 207 | 46 |
| **Brachytherapy** | 116 | 75 | 31 | 20 | 6 |
| **PADT** | 149 | 355 | 447 | 1256 | 985 |
| **Radical prostatectomy** | 309 | 396 | 182 | 484 | 59 |
| **Radical radiotherapy** | 354 | 619 | 671 | 1085 | 536 |
