# Supplementary material for: Improving Clinical Risk Stratification at Diagnosis in Primary Prostate Cancer: A Prognostic Modelling Study
Source: PLoS Med. 2016 Aug 2;13(8):e1002063. doi: 10.1371/journal.pmed.1002063 (PMC4970710; doi:10.1371/journal.pmed.1002063)
Supplement: S4 Table — (DOCX) [file pmed.1002063.s005.docx]

**Table S4** – Comparison of radical therapy use in each new risk group across the whole cohort. Test for homogeneity showed no difference in the rate of uptake between each group (IRR – Incidence rate ratio of radical therapy; either prostatectomy, radiotherapy or brachytherapy) (M-H Mantel-Haenszel).

| **New Risk group** | **IRR** | **[95% Conf. Interval]** | **M-H Weight** |
| --- | --- | --- | --- |
|  |  |  |  |
| 1 | 0.172762 | 0.05 - 0.45 | 14.23 |
| 2 | 0.178322 | 0.08 - 0.34 | 28.21 |
| 3 | 0.10358 | 0.04 - 0.19 | 44.97 |
| 4 | 0.12592 | 0.09 - 0.17 | 172.23 |
| 5 | 0.163352 | 0.1 - 0.25 | 86.30 |
|  |  |  |  |
| Crude | 0.126402 | 0.1 | 0.15 |
| M-H combined | 0.138555 | 0.11 | 0.17 |
