# Supplementary material for: Improving Clinical Risk Stratification at Diagnosis in Primary Prostate Cancer: A Prognostic Modelling Study
Source: PLoS Med. 2016 Aug 2;13(8):e1002063. doi: 10.1371/journal.pmed.1002063 (PMC4970710; doi:10.1371/journal.pmed.1002063)
Supplement: S5 Table — (DOCX) [file pmed.1002063.s006.docx]

**Table S5** – Distribution of the external validation cohort (n=1706) by age, PSA at presentation, biopsy grade and clinical stage. PSA in ng/m.

|  |  |  |  |  | |  |  |  |
| --- | --- | --- | --- | --- | --- | --- | --- | --- |
| **Age (y)** | **n** | **PSA** | **n** | **Biopsy grade** | | **n** | **Stage** | **n** |
|  |  |  |  |  |  |  |  |  |
| **<60** | 321 | **<10** | 711 | **<6** | Prognostic score 1 | 587 | **T1** | 585 |
| **60-69** | 723 | **10-20** | 589 | **3+4** | Prognostic score 2 | 487 | **T2** | 578 |
| **70-79** | 559 | **>20** | 406 | **4+3** | Prognostic score 3 | 210 | **T3** | 537 |
| **≥ 80** | 103 |  |  | **8** | Prognostic score 4 | 192 | **T4** | 6 |
|  |  |  |  | **9-10** | Prognostic score 5 | 230 |  |  |
