# Supplementary material for: Improving Clinical Risk Stratification at Diagnosis in Primary Prostate Cancer: A Prognostic Modelling Study
Source: PLoS Med. 2016 Aug 2;13(8):e1002063. doi: 10.1371/journal.pmed.1002063 (PMC4970710; doi:10.1371/journal.pmed.1002063)
Supplement: S1 Text — (DOCX) [file pmed.1002063.s007.docx]

**Improving clinical risk stratification at diagnosis to better predict cancer specific mortality in primary prostate cancer: A prognostic modelling study.**

**Analysis plan ( 20^th^ March 2016)**

Objective

The primary objective of the study was to develop a new prognostic risk stratification model for prostate cancer specific mortality in men with non-metastatic prostate cancer. The concept of the study was first discussed in the autumn of 2014. The study idea was conceived from previous work done by the group in exploiting a large East of England UK cancer registry cohort to investigate changing presentations and treatment trends in men with prostate cancer (Greenberg *et al* 2013; Greenberg *et al* 2015). This cohort had already been well characterised and clinic-pathological variables and treatment modality established along with survival outcomes. A total cohort of 10,139 men were identified for this current study. As we were using fully anonymised data, there was no requirement to submit the study for Ethics.

Study rationale

The reference model was the 3 strata NICE risk criteria which is itself based on the D’Amico criteria first reported in the 1990s. This model utilises standard clinic-pathological details (histological grade, clinical stage and serum prostate specific antigen levels) available at diagnosis to assign a risk group to a patient: low, intermediate or high-risk. This remains the current standard in UK clinical practice (updated in 2014) to guide clinical decision making. A key problem with this current model is that there is known to be a large heterogeneity in outcomes within the risk groups. Moreover it has never been tested against prostate cancer specific mortality as an outcome. As such, day to day clinical decision making can be difficult and imprecise. The goal of the study was to see if a similarly simple (using the same diagnostic information) but refined model could be constructed with improved prognostic ability. This reference model was used as a basis on which to postulate the new risk model based on 2 notions. (i) A previous observation that the number of risk factors in an individual risk strata could identify better and poor performing subgroups of in high-risk surgically treated men (Joniau *et al* 2015) and (ii) the imminent introduction of a new prognostic based pathological grading system by the International society of uro-pathologists (ISUP) (recent updated publication Epstein *et al* 2016). These prognostic groups had not previously been included in any risk prediction model and it was unclear how they would map on to the NICE risk criteria for future use.

Analysis strategy

A training set and a separate testing set was derived from the larger study cohort by split sample random seed generation. The primary outcome of interest was a priori defined as prostate cancer specific mortality. The initial analysis was based on using cox hazards model, the log rank test and Kaplan Meier curves in the training set and then validation in the testing set. We also assessed model calibration. If the new model showed good discrimination between groups, the analysis would proceed to i) Explore the impact of competing risks of death on model performance and (ii) Quantify the incremental prognostic value of the new model versus the current NICE criteria by calculating and comparing the concordance indices of the 2 models.

External validation cohort

In April 2015, our early results appeared promising and we identified the need for a separate external validation cohort. An approach was made to the Northern Ireland Cancer registry and collaboration was established with Dr Ann Gavin who became a contributor to the paper. This yielded us an external validation cohort of 1706 patients in which to retest our results.

Modification to the analysis following peer review of the manuscript

The new 5 strata model and the analysis undertaken in comparison to the NICE criteria are described and shown in the paper. Following peer review however, additional analysis were suggested by the reviewers and these are included in our paper in a revision. Principal amongst these were:

1. Inter group comparisons rather than comparison against only Group 1 for the competing risks

model.

2. New concordance index testing to include competing risks

3. Model calibration curves with inclusion of competing risks

References relevant to the analysis plan

1. Greenberg DC, Wright KA, Lophathanon A, Muir KR, Gnanapragasam VJ. Changing presentation of prostate cancer in a UK population--10 year trends in prostate cancer risk profiles in the East of England. Br J Cancer. 2013 Oct 15;109(8):2115-20.

2. Greenberg DC, Lophatananon A, Wright KA, Muir KR, Gnanapragasam VJ. Trends and outcome from radical therapy for primary non-metastatic prostate cancer in a UKpopulation. PLoS One. 2015 Mar 5;10(3):e0119494.

3. NICE. Prostate cancer: diagnosis and treatment. NICE guidelines [CG175]. 2014

4. Joniau S, Briganti A, Gontero P, Gandaglia G, Tosco L, Fieuws S, Tombal B, Marchioro G, Walz J, Kneitz B, Bader P, Frohneberg D, Tizzani A, Graefen M, van Cangh P, Karnes RJ, Montorsi F, Van Poppel H, Spahn M; European Multicenter Prostate Cancer Clinical and Translational Research Group (EMPaCT). Stratification of high-risk prostate cancer into prognostic categories: a European multi-institutional study. Eur Urol. 2015 Jan;67(1):157-64. (Epub 2014 Jan 25)

5. Epstein JI, Egevad L, Amin MB, Delahunt B, Srigley JR, Humphrey PA; GradingCommittee. The 2014 International Society of Urological Pathology (ISUP) Consensus Conference on Gleason Grading of Prostatic Carcinoma: Definition of Grading Patterns and Proposal for a New Grading System. Am J Surg Pathol. 2016 Feb;40(2):244-52.
